# Multiplex Assay for Live-Cell Monitoring of Cellular Fates of Amyloid-β Precursor Protein (APP)

**DOI:** 10.1371/journal.pone.0098619

**Published:** 2014-06-16

**Authors:** Maria Merezhko, Pranuthi Muggalla, Niko-Petteri Nykänen, Xu Yan, Prasanna Sakha, Henri J. Huttunen

**Affiliations:** Neuroscience Center, University of Helsinki, Helsinki, Finland; Massachusetts General Hospital, United States of America

## Abstract

Amyloid-β precursor protein (APP) plays a central role in pathogenesis of Alzheimer's disease. APP has a short half-life and undergoes complex proteolytic processing that is highly responsive to various stimuli such as changes in cellular lipid or energy homeostasis. Cellular trafficking of APP is controlled by its large protein interactome, including dozens of cytosolic adaptor proteins, and also by interactions with lipids. Currently, cellular regulation of APP is mostly studied based on appearance of APP-derived proteolytic fragments to conditioned media and cellular extracts. Here, we have developed a novel live-cell assay system based on several indirect measures that reflect altered APP trafficking and processing in cells. Protein-fragment complementation assay technology for detection of APP-BACE1 protein-protein interaction forms the core of the new assay. In a multiplex form, the assay can measure four endpoints: total cellular APP level, total secreted sAPP level in media, APP-BACE1 interaction in cells and in exosomes released by the cells. Functional validation of the assay with pharmacological and genetic tools revealed distinct patterns of cellular fates of APP, with immediate mechanistic implications. This new technology will facilitate functional genomics studies of late-onset Alzheimer's disease, drug discovery efforts targeting APP and characterization of the physiological functions of APP and its proteolytic fragments.

## Introduction

Amyloid-β precursor protein (APP) is multifunctional glycoprotein and a source of several proteolytically generated bioactive peptides [1], [2]. Amyloid-β peptide (Aβ) is a major constituent of amyloid plaques, a pathological hallmark of Alzheimer's disease [3]. In the nervous system, soluble Aβ has prominent inhibitory effects on synaptic function and plasticity [4], [5]. Aβ is derived from amyloid-β precursor protein (APP) via sequential proteolytical cleavages by β-secretase (BACE1) and γ-secretase [6], [7]. Other fragments, such as sAPP-α, sAPP-β and APP intracellular domain (AICD), have paracrine and cell-autonomous regulatory functions, which remain incompletely characterized [8]–[11]. In addition to peptides from the well-characterized α-, β- and γ/ε cleavages, also other cleavage products of APP have been described in specific conditions [12]–[15].

APP has a rapid turnover in most cells types [16], [17], and protelolytic processing plays a central role in APP's lifecycle and functions [2], [18]. While nonamyloidogenic processing of APP by α-secretases takes mostly place at the cell surface, amyloidogenic β- and γ-secretase-mediated processing of APP occurs within intracellular vesicular compartments, especially endosomes [19]–[21]. Therefore, endocytosis and subcellular vesicular trafficking of APP are major determinants of the cellular fate of APP [22]. The APP trafficking and processing system is highly responsive to various aspects of cellular metabolism and stress, including alterations in lipid, Ca^2+^ and energy homeostasis [17], [23]–[27]. A complex interplay of proteins interacting with APP determines its cellular trafficking and fate. Particularly, there seems to be a large number of cytosolic adaptor proteins that interact with the cytosolic domain of APP and regulate its internalization and further subcellular trafficking [28]. Moreover, interaction with membrane lipids also modulates APP trafficking and processing [29], [30].

A variety of methods have been applied for studying the cell biology of APP. Western blot and ELISA are the standard methods to study proteolytic fragments of APP. In addition, techniques like affinity capture-mass spectrometry offer an unbiased way for characterization of components of the APP interactome [28], [31], [32], while fluorescence resonance energy transfer (FRET)-based techniques have proven useful for visualization and studying dynamics of individual protein-protein interactions based on physical proximity in living cells [33], [34]. Studying the complex cellular regulation of APP, especially the dynamic features, would benefit from development of novel tools that can be used in live cells, preferably with high-throughput capacity.

Among the more than 200 currently known protein interactors of APP [28], BACE1 has attracted perhaps the most attention [35]. Since BACE1-mediated amyloidogenic cleavage of APP is the rate-limiting step in generation of Aβ peptide, better understanding of the dynamics of APP-BACE1 interaction could help explain cellular mechanisms involved in the early stages of pathogenesis of Alzheimer's disease. Moreover, the normal physiological function(s) of APP and its proteolytic fragments remain poorly understood. Better understanding of the molecular-level regulation of APP-BACE1 interaction can provide novel insight into the cellular functions of these proteins. Here, we have developed a novel approach for studying APP-BACE1 interaction using a protein-fragment complementation assay (PCA) based on the small Gaussia princeps luciferase (GLuc) [36]. This sensitive APP-BACE1 protein-protein interaction assay was combined with alkaline phosphatase-based detection of secreted sAPP fragments providing a four-readout multiplexed assay platform capable of delivering mechanistic details on how APP is regulated in live cells.

## Materials and Methods

### DNA plasmid construction

The original humanized *Gaussia princeps* PCA plasmids [36] were donated by Dr. Stephan Michnick (Université de Montréal, Canada) and were constructed in the pcDNA3.1/zeo (Invitrogen) backbone. The hGLuc-tagged APP_695_ constructs were generated and donated by Dr. Oksana Berezovska (Massachusetts General Hospital, Boston, MA). All APP constructs used in this study expressed the neuronal APP_695_ isoform lacking the KPI domain. The cDNA of BACE1 was a gift from Dr. Dora Kovacs (Massachusetts General Hospital, Boston, MA). For APP and BACE1 (both type 1 transmembrane proteins), the hGLuc fragment was placed in the cytosolic C-terminus after a (GGGGS)_2_SG linker. The APP-hGLuc constructs were further modified by fusing the secreted alkaline phosphatase (AP) fragment to the N-terminus replacing the endogenous APP signal peptide [pEAK12-AP/APP plasmid was a kind gift from Dr. Stephan Lichtenthaler (Ludwig-Maximilians-Universität München, Germany)] [37]. The identity of all constructs was confirmed by DNA sequencing.

### Cell culture, transfection and RNAi

Mouse Neuro-2A (N2A) neuroblastoma cells (ATCC) were cultured in DMEM supplemented with 10% (v/v) FBS (Invitrogen), 1% (v/v) L-glutamine-penicillin-streptomycin solution (Lonza) at 37°C in a water-saturated air, 5% CO_2_ atmosphere. N2A cells were transfected with JetPEI (Polyplus) according to the manufacturer's instructions. The transfection conditions were optimized so that on average at least 80% transfection efficiency was reached. Gene silencing was achieved by co-transfection of plasmids encoding shRNA for mouse GGA3 or mouse VPS35 (OpenBiosystems/Thermo Scientific). Gene silencing efficiency was tested in N2A cells using Western blotting.

### Protein fragment-complementation assay

N2A cells were plated on poly-L-lysine-coated white-wall 96-well plates (PerkinElmer Life Sciences). 125 ng of plasmid DNA was used for transient transfection per well, divided as follows: 58.75 ng of GLuc1 reporter plasmid, 58.75 ng of GLuc2 reporter plasmid, and 7.5 ng of pRC/CMV-β-galactosidase (βGal) as an internal vector control. In case of co-transfection with shRNA plasmids, the ratios were as follows: GLuc1 27 ng, GLuc2 27 ng, shRNA 40 ng and βGal 6 ng. Experiments were carried out 48 h after transfection with four replicate wells used per experimental condition. Briefly, the cells were washed with warm PBS and placed in phenol red-free DMEM (Invitrogen) without serum. Test compounds were added to the medium, and the cells were incubated for 2–24 h. Brefeldin A (BFA) and Bafilomycin A1 were purchased from Sigma. BACE inhibitor IV was obtained from Calbiochem and DAPT was from Biomol (Enzo Life Sciences). Dynole 34-2 was purhcased from Tocris/R&D Systems. DMSO was used as a vehicle control. For detection of the hGLuc PCA signal, the cells were exposed to well-by-well injections of 25 µl (final concentration, 20 µM) of native coelenterazine (Nanolight Technology), and the emitted luminescence was detected immediately by flash luminometry using a Victor^3^ plate reader (PerkinElmer Life Sciences). Individual experiments were repeated three or four times. After measuring the PCA signal, cells were lysed for βGal assay with 40 µl of lysis buffer/well (120 mM Tris-HCl, pH 7.5, 120 mM NaCl, 6 mM MgSO_4_, 6% Triton X-100) and incubated for 30 min at room temperature with mild shaking. Then, 45 µl of βGal substrate o-nitrophenyl-β-D-galactopyranoside (Sigma, 4 mg/ml stock in sterile water) solution was combined with 55 µl of cleavage buffer (120 mM sodium phosphate, pH 7.0, 24 mM KCl, 2.4 mM MgSO4, and 2.4 mM DTT), and 100 µl of this solution was added per well. The plates were incubated at +37°C for a further 30 minutes and absorbance was read at 405 nm.

### Secreted alkaline phosphatase (SEAP) assay

Conditioned media was collected at indicated time points and clarified by centrifugation at 5,000× g for 10 min. Secreted sAPP was measured by a chemiluminescent Secreted Alkaline Phosphotase reporter gene assay (SEAP Assay; Roche Applied Science) according to the manufacturer's instructions.

### Multiplex PCA

PCA part was adjusted by changing the ratios of plasmids for transient transfection. 125 ng of plasmid DNA was divided as follows: 33.75 ng of GLuc1 reporter plasmid, 83.75 ng of GLuc2 reporter plasmid, and 7.5 ng of βGal plasmid. 42 h after transfection, the cells were washed with warm PBS and placed in 130 µl of phenol red-free DMEM (Invitrogen) without serum. Test compounds were added to the medium, and the cells were incubated for 6 h. Media was collected and cleared by centrifugation at 5,000× g for 10 min. The cells were placed in fresh phenol red-free DMEM without serum and hGLuc PCA signal was detected as described above. After measuring the PCA signal, cells were lysed for βGal assay as described above. 25 µl of lysates were collected to measure total cellular APP by SEAP assay. Then, βGal assay was carried out as in a single-readout PCA. Supernatant was split in 2 samples and analyzed separately: 25 µl of media was used to measure shed sAPP by SEAP assay and 75 µl was used to measure exosomal APP-BACE1 by PCA. PCA and SEAP assay signals were normalized to βGal signals well by well.

### Western blots

For Western blots, cells were transfected on 6-well polystyrene plates (Corning). 24 h after transfection, the cells were washed twice with ice-cold PBS followed by scraping and extraction on ice for 30 min in a buffer containing 10 mM Tris-HCl, pH 6.8, 1 mM EDTA, 150 mM NaCl, 0.25% Nonidet P-40, 1% Triton X-100, 1 µM NaF and protease inhibitor mixture tablets (Roche Applied Science). Cell debris was removed by centrifugation at 16,000× g. The protein concentrations were determined using the BCA protein assay kit (Pierce/Thermo) and equal amounts of total protein (25–40 µg) per lane were resolved in a 4–12% gradient Bis-Tris gels (Novex, Invitrogen) under reducing conditions. After transfer to PVDF membranes (Amersham Biosciences/GE Healthcare), the filters were probed with the following antibodies: A8717 (Sigma; APP C-terminus), dNGluc (Proteintech Group Inc) and GAPDH (Millipore). After incubation with horseradish-conjugated secondary antibodies, the signal was developed using ECL Western blotting detection reagent (Pierce/Thermo).

### Isolation of exosomal vesicles

N2A cells were plated on 10-cm plates with full DMEM. Cells were transfected at about 50% confluency. Cells were washed once with pre-warmed PBS and placed in serum-free, phenol red-free DMEM. 24 h later, the conditioned media was collected for isolation of exosomes, and the cell extracts were prepared for Western blot analysis. Conditioned media (pooled from 6 10-cm plates) was centrifuged successively at 200× g for 10 min to remove floating cells, at 2,000× g for 10 min to remove dead cells, at 10,000× g for 30 min at 4°C to remove the cell debris. After each step, the supernatant was recollected without disturbing the pellets. The cell- and debris-free supernatant was centrifuged using a Beckman Optima L-100K centrifuge and an SW41 Ti rotor at 100,000× g for 70 min at 4°C to collect the exosomes. The supernatant was gently removed and the exosomal pellet resuspended in 1 ml of PBS. The collected PBS was centrifuged again at 100,000× g for 70 min at 4°C. After careful removal of the PBS supernatant, the exosomal pellet was resuspended in 40 µl of 1.5 X Laemmli buffer (75 mM Tris-HCl, pH 6.8, 3% SDS, 15% glycerol, 3.75 mM EDTA) with β-mercaptoethanol, and analyzed by Western blotting.

### Aβ ELISA

For Aβ determination, N2A cells were transfected with APP-GLuc2 plasmid on 6-well plates (Corning), and 24-h conditioned media (serum-free) collected 48 h post-transfection. The conditioned media was cleared from debris and secreted Aβ_40_ and Aβ_42_ were quantitated by standard sandwich ELISA (pmol/L; IBL International GmbH, Germany). Protein concentrations of the cell lysates were determined using the BCA protein assay kit (Pierce). The Aβ values were normalized to total protein levels (mg). Each experiment was carried out in triplicate.

### Statistical analyses

Statistical analyses were performed using analysis of variance (ANOVA; three or more groups, followed by Bonferroni's post-tests) or Student's t test (two groups) in GraphPad Prism software. Significance was placed at p<0.05.

## Results

### Protein-fragment complementation assay-based detection of APP-BACE1 interaction in live cells

**Figure 1:**
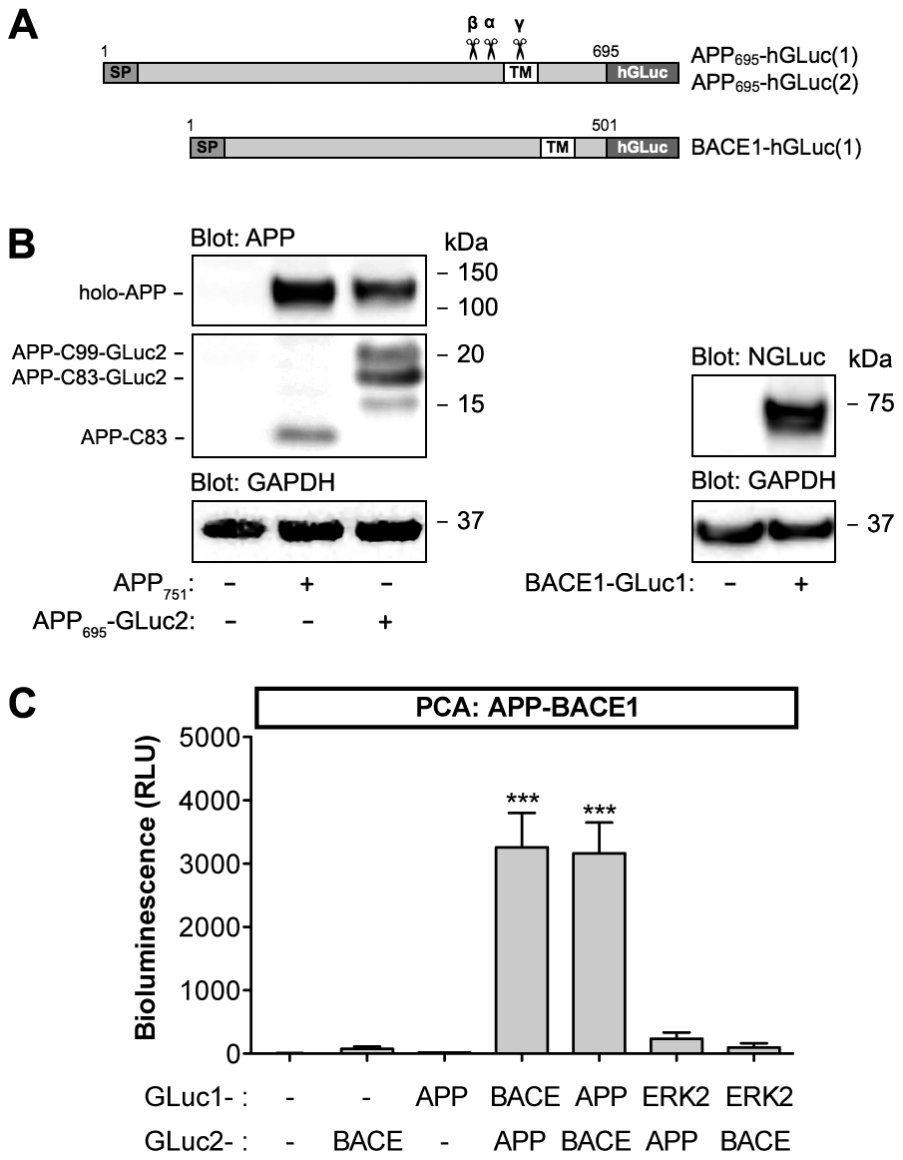
Protein-fragment complementation assay-based detection of APP and BACE1 interaction in live cells. (A) Graphical presentation of PCA reporter constructs of APP and BACE1. hGLuc, humanized *Gaussia* luciferase fragment; SP, signal peptide; TM, transmembrane domain. (B) Normal expression and proteolytic processing of APP-GLuc2 and BACE1-GLuc1 fusion proteins in N2A neuroblastoma cells. Cells were transiently transfected with indicated combinations of expression constructs and analyzed for APP fragments and BACE1 protein in cell lysates. Western blots were probed with APP C-terminal antibody (A8717), dNGluc antibody (detects the GLuc1 fragment) and GAPDH as a loading control. (C) Validation of GLuc-based PCA for detection of APP-BACE1 interaction in N2A cells. Cells were transiently transfected with indicated combinations of expression constructs. Luminescence signal was measured 48 h post-transfection in live cells. Normalization of cell numbers and transfection efficiency was done with an internal vector control (using a plasmid expressing β-galactosidase). Control plasmids were empty GLuc1/2 plasmids expressing the indicated GLuc fragment alone. The values are normalized bioluminescence signals recorded from expressed pairs of reporter constructs. Error bars represent the SEM, and statistical significance was assessed using ANOVA. *** p<0.001.

Because of its pathophysiological significance, we focused on APP-BACE1 protein-protein interaction as the first readout of the new assay system. Split luciferase PCA technology offers a suitable experimental strategy for developing a live-cell assay for studying APP-BACE1 interaction dynamics. We chose a PCA based on a small Gaussia princeps luciferase (GLuc) reporter, as it provides a highly sensitive and reversible detection method for protein-protein interactions [36]. Humanized GLuc protein without the 16 N-terminal amino acids was split between the Gly93 and Glu94 residues, resulting in a 93 amino-acid (10.1 kDa) GLuc1 fragment and a 76 amino acid (8.2 kDa) GLuc2 fragment. Since APP and BACE1 are both type 1 transmembrane proteins with relatively short cytosolic domains, we placed GLuc1 and GLuc2 tags in the C-termini of APP and BACE1 in both orientations (Fig. 1A). In order to maximize the topological flexibility of the GLuc fragments, (GGGGS)_2_SG linker region was placed between the C-terminus of the protein of interest and the GLuc fragments. GLuc-tagged proteins were expressed normally in N2A cells as shown by the Western blots (Fig. 1B). The GLuc-tags did not interfere with proteolytic processing pattern of APP, as APP-GLuc1 and APP-GLuc2 fusion proteins produced α- and β-C-terminal fragments similarly to non-tagged APP (Fig. 1B).

When single GLuc-tagged APP and BACE1 constructs were expressed alone in N2A (with control plasmids expressing free GLuc1/2 reporter fragments), only very low background luminescence signal was detected when the cells were exposed to native coelenterazine, the substrate of Gaussia luciferase (Fig. 1C). When APP-GLuc1 was co-transfected with BACE1-GLuc2 plasmid, a robust luminescence signal was detected (Fig. 1C). Similar signal was detected from cells co-expressing the APP-GLuc2 and BACE1-GLuc1. To control for specificity of APP-BACE1 PCA, we tested APP-GLuc2 and BACE-GLuc2 interaction with GLuc1-tagged extracellular signal-regulated kinase 2 (ERK2), a mitogen-activated protein kinase for which direct interaction with APP or BACE1 has not been reported. As shown in Fig. 1C, there was very low PCA signal generated by APP-ERK2 or BACE1-ERK2 suggesting that there was no direct interaction between these proteins.

**Figure 2:**
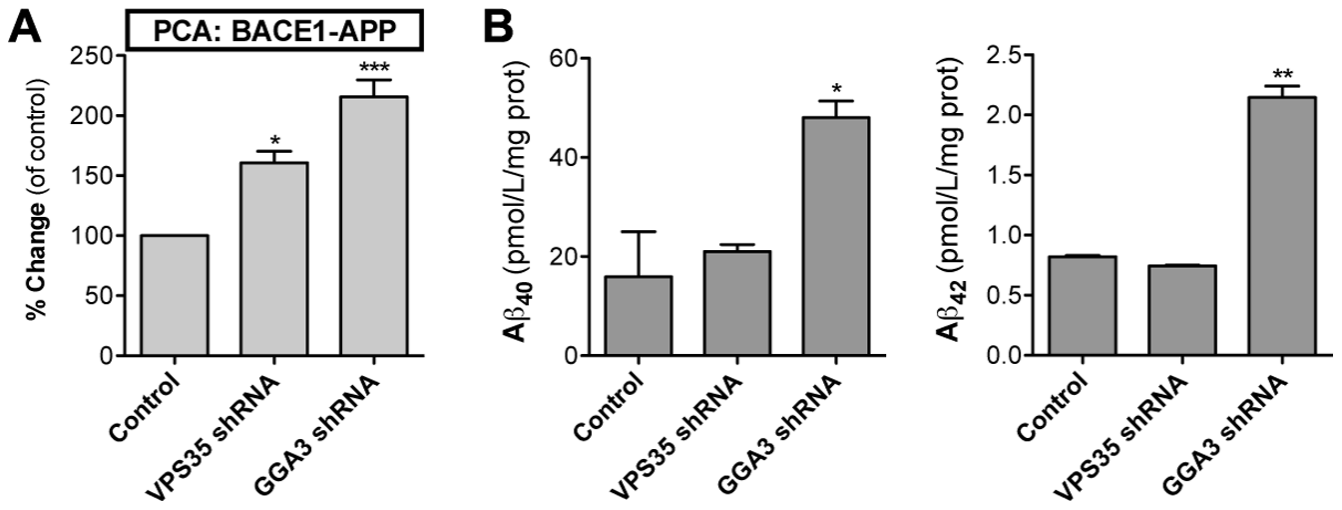
Functional assay validation of GLuc PCA for APP-BACE1 interaction. For genetic assay validation of BACE1-APP PCA, plasmids expressing GGA3 and VPS35 shRNA were cotransfected to N2A cells with plasmids encoding BACE1-GLuc1 and APP-GLuc2 reporters. (A) PCA signal was measured at 48 h post-transfection. (B) Aβ_40_ and Aβ_42_ in conditioned media were determined by sandwich ELISA. The number of replicate wells for PCA was four (96-well plate) and for Aβ ELISA two (6-well plate). Error bars represent the SEM, and statistical significance was assessed using ANOVA. * p<0.05, ** p<0.01, *** p<0.001.

Next, we tested the dynamics of the APP-BACE1 interaction assay using genetic means, based on previously established regulators of APP metabolism. Previous studies have shown that GGA3 is an important regulator of BACE1 trafficking from endosomes for lysosomal degradation [38]. Depletion of GGA3 stabilizes BACE1 protein increasing its levels in the endocytic compartment and therefore results in increased Aβ generation. Similarly, knockdown of VPS35, a retromer receptor involved in endosome-Golgi trafficking of APP, was previously reported to promote Aβ generation [39]. Thus, we decided to use GGA3 and VPS35 RNAi as the validation tool in BACE1-APP PCA assay development. The knockdown efficiencies of GGA3 and VPS35 shRNA were determined by Western blotting. As compared to control cells, the expression of the corresponding shRNA's in N2A cells reduced GGA3 protein level by −62% and VPS35 by −53% (data not shown). When the GGA3 and VPS35 shRNA plasmids were co-transfected with the PCA plasmids, the BACE1-APP PCA signal was increased by 216% in GGA3 shRNA cells (Fig. 2A). This indicates that GGA3 knockdown increases interaction between BACE1 and APP. To confirm that the increased interaction correlates with an increase in Aβ production, we analyzed conditioned media from cells expressing the BACE1-APP PCA constructs and shRNA plasmids. Aβ_40_ and Aβ_42_ levels in the conditioned media were increased by 301% and 262% in GGA3 shRNA cells, respectively (Fig. 2B), confirming that the increased interaction measured by the PCA resulted in increased Aβ generation. VPS35 shRNA cells did not show significant increases in Aβ secretion although the BACE1-APP PCA signal was increased by 161% in VPS35 shRNA cells. These data show that GLuc PCA can detect changes in APP-BACE1 protein interaction in live cells.

### Simultaneous live-cell detection of BACE1-APP interaction and shedding of APP

**Figure 3:**
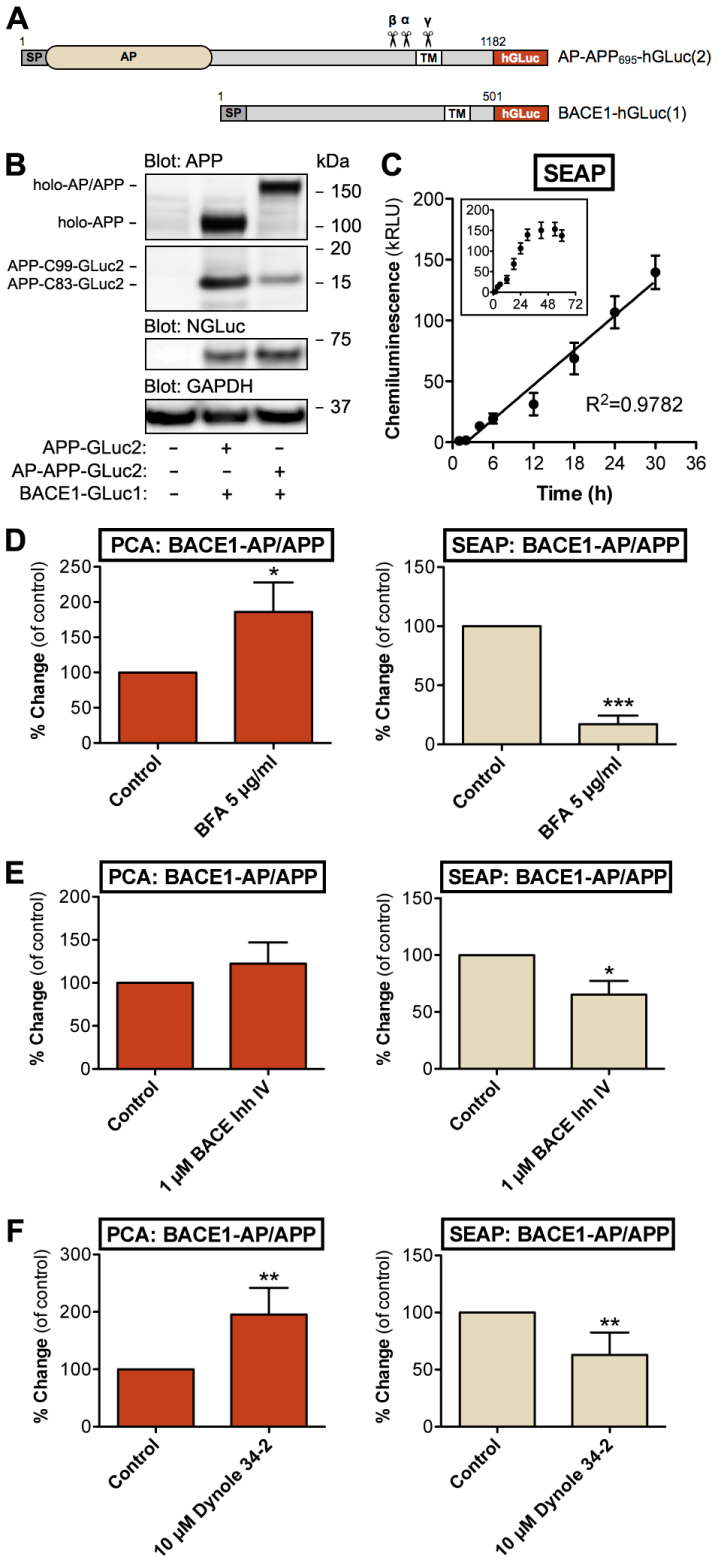
Multiplex assay for simultaneous live-cell detection of APP-BACE1 interaction and proteolytic cleavage of APP. (A) Graphical presentation of the multiplex PCA reporter constructs. Alkaline phosphatase (AP) with a signal peptide was placed in the N-terminus of APP-GLuc2. AP reporter is depicted in beige and GLuc reporter in red color. The same colors are used in column graphs in Fig. 3B–F for corresponding reporter data. (B) Normal proteolytic processing of the AP/APP-hGLuc fusion protein in N2A cells. Cells were transiently transfected with indicated combinations of various APP constructs: APP-GLuc2 and AP/APP-GLuc2 and BACE1-GLuc1. Western blots were probed with APP C-terminal antibody (A8717), dNGluc (BACE1) and GAPDH as a loading control. (C) Sensitivity and linearity of secreted alkaline phosphatase-sAPP (SEAP) assay from conditioned media. N2A cells were transiently transfected with BACE1-GLuc1 and AP/APP-GLuc2. Cells were incubated in serum-free media for up to 30 hours (inset graph shows data up to 60 h). AP activity in cell-free conditioned media was detected using a chemiluminescent SEAP assay. Normalization of cell numbers and transfection efficiency was done with an internal vector control (using a plasmid expressing β-galactosidase). The values are normalized chemiluminescence signals recorded from expressed pair of constructs. The number of replicate wells was four. Linearity of data was evaluated by regression analysis; correlation coefficient (R^2^) was 0.98806. Error bars represent the SEM. (D) Effects of brefeldin A (BFA) in the multiplex assay (PCA+AP data). N2A cells were transiently transfected with BACE1-GLuc1 and AP-APP-GLuc2, and treated with indicated concentration of BFA for 24 h before measurement of PCA and SEAP signals (48 h after transfection). The average values are displayed as percentage of change as compared to vehicle-treated control cells. (E) Effects of BACE inhibitor IV in the multiplex assay (PCA+AP data). N2A cells were transfected as in D, and treated with indicated concentration of BACE inhibitor IV for 6 h before measurement PCA and SEAP signals (48 h after transfection). The average values are displayed as percentage of change as compared to vehicle-treated control cells. (F) Effects of dynole 34-2 in the multiplex assay (PCA+AP data). N2A cells were transfected as in D, and treated with indicated concentration of dynole 34-2 for 6 h before measurement PCA and SEAP signals (48 h after transfection). The average values are displayed as percentage of change as compared to vehicle-treated control cells. Error bars represent the SEM, and statistical significance was assessed using Student's t test (four replicate wells/experiment, four independent experiments). * p<0.05, ** p<0.01, *** p<0.001.

Since APP ectodomain release by proteolytic cleavage is the expected outcome of the BACE1-APP interaction, we sought for ways to combine detection of proteolytic processing of APP with the live-cell PCA assay. Alkaline phosphatase (AP) fused to the N-terminus of APP (after signal peptide) has been previously used to detect secreted APP (sAPP) fragments from the conditioned media [37]. For simultaneous detection of BACE1 interaction and proteolytic cleavage of APP, we generated a double-fusion construct of APP, which contains an N-terminal AP fusion and a C-terminal GLuc-fusion (Fig. 3A). This allows using a sensitive luminescence-based assay (SEAP = secreted alkaline phosphatase assay) for detection of sAPP fragments from the conditioned media. The AP-APP-GLuc2 was expressed and processed normally in N2A cells, as compared to the APP-GLuc2 construct that does not have the AP fusion (Fig. 3B). Next, accumulation of shed sAPP from cleared, conditioned media was analyzed. SEAP assay detected sAPP from media as early as 2 h after media change, and showed a linear curve of sAPP production at least up to 18 hours (Fig. 3C; R^2^ = 0.9782).

Optimally the shedding assay would detect only sAPP-β fragments derived from BACE1-mediated cleavage of APP. In order to make the assay specific for analysis of sAPP-β, we tested blocking of α-secretase-mediated processing of APP via genetic means. We introduced the F615P mutation (according to APP_695_ numbering) close to the α-secretase cleavage site in the AP/APP-GLuc2 construct. This mutation was previously reported to reduce sAPP secretion by >60% [40]. We observed only a minor shift towards β-cleavage, not a complete blockage of α-processing (data not shown). This observation was supported by a recent study showing that F615P mutation can only partially inhibit α-secretase cleavage of APP [41]. Therefore, we decided that the APP cleavage assay readout shall be the total sAPP (sAPP-α+sAPP-β) in media as determined by the SEAP assay.

Next, we used pharmacological tools to functionally validate the two-parameter assay. Brefeldin A (BFA) inhibits the transport of secretory and membrane proteins from the ER to the Golgi. Retention of APP in the early secretory pathway has been reported to reduce the proteolytic processing of APP and shedding of sAPP fragments to media [17], [42]. In line with the previous reports, treatment of cells with BFA strongly reduced sAPP shedding (Fig. 3D; SEAP signal in the media). Interestingly, BFA increased the interaction between APP and BACE1, likely due to their accumulation in the ER (Fig. 3D, PCA data on the left panel). Inhibition of β-cleavage of APP by BACE inhibitor IV, a BACE1 active-site binding compound, reduced the amount of total secreted sAPP in media by 35% while increasing the APP-BACE1 interaction by 22% (Fig. 3E). This suggests that inhibitor-bound BACE1 molecules can still interact with APP although the actual enzymatic cleavage of APP is inhibited.

Endocytosis is required for internalization of APP and BACE1 from the cell surface [43]–[45] to the endocytic compartment where BACE1 activity is increased by acidification of the vesicle lumen [35]. Inhibition of endocytosis reduces APP internalization and reduces Aβ production in cell lines [19], [46]. Dynole 34-2, a cell-permeable small-molecule inhibitor of dynamin-dependent endocytosis [47], significantly inhibited sAPP secretion (−37%) but increased APP-BACE1 interaction by 95% (Fig. 3F). This pattern is similar to BACE1 inhibition and suggests that conditions interfering with internalization of APP and BACE1 or inhibition of BACE1 activity increase the interaction between the two proteins although BACE1-mediated by proteolytic processing of APP is reduced. Moreover, these data show that the two-readout multiplex assay of AP/APP-BACE1 interaction and APP shedding responds to known modulators of APP-BACE1 trafficking and processing in a predictable manner.

### Exosomal secretion of APP and BACE1 PCA reporter proteins

**Figure 4:**
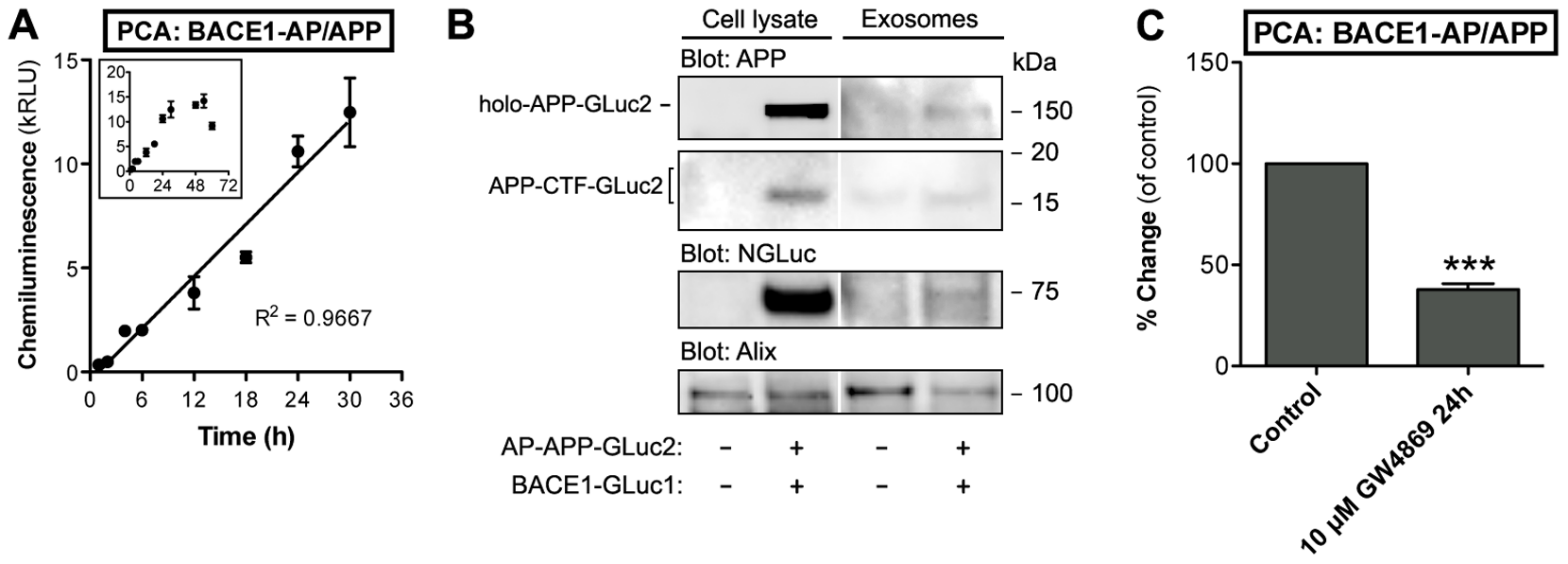
Exosomal secretion of AP/APP and BACE1 PCA reporter proteins. (A) Detection of AP/APP-BACE1 PCA signal in cleared, cell- and debris-free conditioned media. N2A cells were transiently transfected with BACE1-GLuc1 and AP/APP-GLuc2. Cells were incubated in serum-free media for up to 18 hours. PCA signals in cell-free conditioned media were detected at indicated time points. Normalization of cell numbers and transfection efficiency was done with an internal vector control. The values are normalized bioluminescence signals recorded from expressed pair of constructs. The number of replicate wells was four. The linearity was evaluated by regression analysis, correlation coefficient (R^2^) is 0.9971. Error bars represent the SEM. (B) N2A cells were transfected with AP/APP-GLuc2 and BACE1-GLuc1 reporter plasmids. Exosomes were isolated from 30-h conditioned media (inset graph shows data up to 60 h) by ultracentrifugation and the presence of AP/APP-GLuc2 and BACE1-GLuc1 reporters in exosomes was analyzed by Western blotting using antibodies for APP (C-terminal antibody A8717), dNGluc (BACE1) and Alix as an exosomal marker. Total cell extracts were analyzed in parallel with the isolated exosome fraction. (C) Pharmacological modulation of ceramide levels alters exosomal secretion of AP/APP-BACE1 PCA reporters. N2A cells were transiently transfected with BACE1-GLuc1 and AP/APP-GLuc2, and treated with 10 µM of GW-4869, the neutral sphingomyelinase (nSMase) inhibitor, for 24 h. PCA signal in cell-free conditioned media was measured at 48 h after transfection. Normalization of cell numbers and transfection efficiency was done with an internal vector control. The average values are displayed as percentage of change as compared to vehicle-treated control cells. Error bars represent the SEM, and statistical significance was assessed using Student's t test (four replicate wells/experiment, four independent experiments). *** p<0.001.

One potential complicating factor for interpretation of the SEAP assay signal from conditioned media could be the presence of uncleaved APP holoprotein in the media. Exosomal release of APP holoprotein, its metabolites and BACE1 by neuronal cells in vitro and in vivo has been reported [48]. Also, overexpression may result in enhanced exosomal release of reporter proteins. Therefore, it is possible the SEAP assay signal represents the combined pool of shedded sAPP and exosomal APP holoprotein, although the exosomal fraction of APP holoprotein is likely to be small as compared to the sAPP fraction. In order to monitor this, we analyzed cell-free conditioned media for the presence of AP/APP-BACE1 complex, as determined by the AP/APP-BACE1 PCA. As shown in Fig. 4A, there is a robust accumulation of AP/APP-BACE1 PCA signal in cleared, conditioned media that showed good linearity at least up to 18 hours (R^2^ = 0.9667). N2A cells constitutively release exosomes enriched in Alix, a multivesicular body protein commonly used as an exosomal marker [49]. To confirm that the AP/APP-BACE1 PCA reporters are found in exosomes, we isolated exomes from the conditioned media using the widely used method described by Théry et al [50]. As shown in Fig. 4B, both AP/APP-GLuc2 and BACE1-GLuc1 PCA reporters were found in Alix-positive exosomes purified from the media using the serial ultracentrifugation method.

To verify that the presence of AP/APP-BACE1 PCA signal in the conditioned media is associated with exosomal secretion, we tested if inhibition of exosome biogenesis would decrease the amount of APP-BACE1 PCA signal in the conditioned media. Sphingolipid metabolism, ceramide in particular, is an important regulator exosome biogenesis. Ceramide is the key regulator of cargo segregation into distinct subdomains of the endosomal membrane before budding of exosomal vesicles into multivesicular endosomes [51]. Inhibition of neutral sphingomyelinase (nSMase), which converts sphingomyelin to ceramide, by GW-4869 significantly inhibited accumulation of AP/APP-BACE1 PCA signal in the conditioned media (−62%; Fig. 4C). These data suggest that 1) both APP and BACE1 holoproteins can be released from cells in association with exosomes, and 2) that AP/APP-BACE1 PCA in the conditioned media can be used as a readout of the exosomal release activity of these proteins. Thus, analysis of exosomal secretion of APP and BACE1 adds another multiplexing dimension for analyzing APP trafficking in live cells.

### Four-parameter multiplex assay for analyzing cellular fate of APP

**Figure 5:**
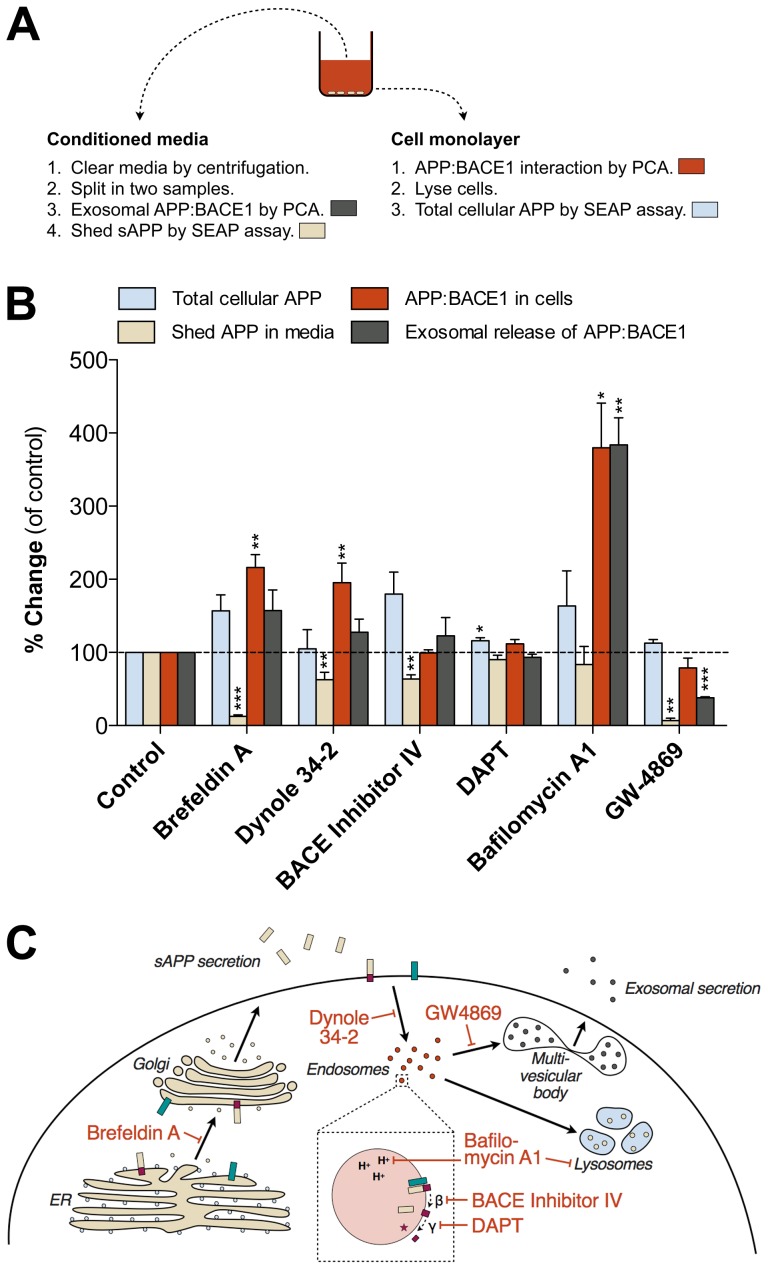
Four-parameter multiplex assay for detection of cellular fates of APP. (A) Workflow and separation of samples for different analyses in the four-endpoint multiplex assay. Colors refer to the parameter/analyte used in the multivariate analysis graph in Fig. 5B. PCA and SEAP signals were read from both conditioned media and cell monolayer (in fresh media) and represent the presence of BACE1-APP complex and APP/sAPP species in different compartments. (B) Multivariate analysis of PCA and SEAP data from cells treated with brefeldin A, dynole 34-2, BACE1 inhibitor IV, DAPT, bafilomycin A1 and GW-4869. N2A cells were transiently transfected with BACE1-GLuc1 and AP/APP-GLuc2, and treated with indicated chemicals for 6 h (1 µM BACE inhibitor IV, 0.5 µM DAPT, 10 µM Dynole 34-2) or 24 h (100 nM bafilomycin, 5 µg/ml brefeldin A, 10 µM GW-4869) before measurement of PCA and SEAP signals (48 h after transfection). The average values are displayed as percentage of change as compared to vehicle-treated control cells. Error bars represent the SEM, and statistical significance was assessed using ANOVA (four replicate wells/experiment, four independent experiments). * p<0.05, ** p<0.01, *** p<0.001. (C) Schematic diagram showing the sites of action of the chemicals used in multiplex PCA in Fig. 5B. Brefeldin A inhibits the transport of secretory and membrane proteins from the ER to the Golgi. Bafilomycin A1, an inhibitor of vacuolar proton ATPases, prevents intravesicular acidification. Dynole 34-2 is an inhibitor of dynamin-dependent endocytosis. GW-4869, neutral sphingomyelinase inhibitor, reduces the exosomal secretion. BACE inhibitor IV and DAPT inhibit β- and γ-secretase cleavage of APP, respectively.

Separation of conditioned media from the cell monolayer allows parallel analysis of multiple readouts. In addition to detection of APP-BACE1 interaction in cells and in secreted exosomes, and total sAPP in media, we used cell-based SEAP signal as a readout of total cellular APP levels. Although a small fraction of the cell-derived SEAP signal likely comes from intracellular sAPP fragments, the vast majority of the signal is derived from APP holoprotein (mature and immature). This is also supported by previous reports [52]. Fig. 5A summarizes the workflow and analyses of different samples in the four-parameter multiplex assay. The average interassay variation was determined for each assay readout by the Coefficient of Variation (%CV), the standard deviation across multiple independent assays expressed as a percentage of the mean (in AP-APP-Gluc2/BACE-GLuc1 transfected cells with no experimental treatments). For total cellular APP (SEAP) %CV was 12.5±2.5%, for shed APP in media (SEAP) 17.1±4.8%, for APP-BACE1 interaction in cells (PCA) 9.9±3.2% and for exosomal APP-BACE1 complexes in media (PCA) 12.2±0.9% (n = 4 replicate experiments). This variation is acceptable for a cell-based assay using transient transfection, according to the widely used criteria in high-throughput assay development (well-to-well %CV should be less than 20%) [53].

We used pharmacological tools previously shown to affect cellular trafficking of APP differentially to test the responsiveness of the four-parameter multiplex APP assay. In addition to compounds used in Figs. 3–4, we added three more compounds for further functional validation. DAPT, a γ-secretase inhibitor [54], acts downstream of BACE1 in the sequential APP proteolytic processing cascade, and is thus not expected to have significant effects in the four-parameter multiplex assay, that mostly focuses on events that operate upstream of γ-secretase. Bafilomycin A1, a vacuolar H^+^-ATPase inhibitor, reduces amyloidogenic processing of APP [55]. Interestingly, bafilomycin A1 also increases cellular release of exosomes [56], likely to compensate for the reduced autophagic/lysosomal degradative capacity in the cells.

The compiled data in Fig. 5B show that the four-parameter multiplex assay responds to all tested experimental conditions in a predictable way, and that the cellular fates of APP in different conditions are quite distinctive. For clarity, the sites of action of the validation compounds are summarized in Fig. 5C. Brefeldin A strongly reduces sAPP secretion due to its effects on early secretory pathway trafficking. BFA results in accumulation of APP and BACE1 to the ER, which in this assay is reflected as an increase in APP-BACE1 interaction. BACE inhibitor IV and dynole 34-2 modulate the BACE1-cleavage of APP via different mechanisms and have somewhat similar effects on the four assay readouts. Inhibition of γ-secretase by DAPT shows little effects on the four readouts. Bafilomycin A1 has the strongest overall effects on APP and BACE1 underscoring its strong impact on endocytic function and lysosomal/autophagic clearance of proteins. Modulation of sphingomyelin-ceramide conversion by GW-4869 not only affects exosomal secretion of APP-BACE1 complex but also strongly reduces sAPP shedding. Cellular levels of ceramide modulate the function of lipid rafts [57], and may also directly affect β-cleavage of APP via altered stability of BACE1 protein [58]. Altogether, these data show that modulation of different sites of the cellular trafficking machinery of APP and BACE1 produces distinctive patterns that can be effectively recognized by the new live-cell assay system.

## Discussion

Here we describe a high-throughput capable live-cell assay system that can rapidly give mechanistic insight into how cellular trafficking of APP is altered by a given perturbation. The assay was developed around live-cell detection of APP-BACE1 protein-protein interaction. The final outcome of the APP-BACE1 interaction – whether APP is cleaved by BACE1 or not – is determined by a wide range of factors. Various subcellular trafficking events determine when, where and for how long APP and BACE1 are allowed to interact within the cell [22]. Moreover, the local microenvironment where the interaction takes place determines whether BACE1 is able to cleave APP, as for example lower intravesicular pH is required for optimal BACE1 enzyme activity [35].

The APP interactome is very large, with many cytosolic domain interactions that regulate its subcellular trafficking and proteolytic processing [28]. New insights into the cellular regulation of the interactions and trafficking of APP can improve our understanding of normal physiological functions of APP and the cellular rationale behind its complex proteolytic processing machinery. Better understanding of the cellular fates of APP can provide new information on how genetic and environmental factors contribute to AD pathogenesis, and offer novel ways for restoring disease pathology to the normal cellular state, e.g. via modulation of key protein-protein interactions of APP. The outcome of our four-parameter multiplex live-cell assay is a distinct fingerprint of the cellular fate of APP, with immediate mechanistic insight, upon changing pharmacological, genetic or metabolic conditions. This feature combined with the high-throughput nature of the assay sets this new technique clearly apart from the existing methods to study cellular regulation of APP.

Expectedly, majority of the proof-of-concept data produced using the novel live-cell APP multiplex assay is in line with previously published reports in the field. Inhibition of the vacuolar H+-ATPase by bafilomycin A1 produced the most robust increase in APP-BACE1 interaction, likely due to reduced efficiency of BACE1-mediated APP cleavage in endosomes with elevated intraluminal pH [55], [59]. γ-secretase inhibition by DAPT had minor effects on the overall pattern of APP interactions and α/β-secretase processing, although a minor and consistent increase in total cellular APP was observed. Similar observations have been reported previously [60].

Genetic manipulation of proteins involved in vesicular trafficking of APP and BACE1, such as GGA3 and VPS35, increased APP-BACE1 interaction, which is in line with previous reports [38], [61]. However, only GGA3 silencing had a strong effect on Aβ levels. There are controversial reports on how VPS35 silencing affects Aβ secretion; both increase [39] and decrease [62] of Aβ40 generation has been reported. These discrepancies may be explained by differences between cell lines or other experimental conditions, such as transfection and gene silencing efficiency, used in various studies. Moreover, in addition to its role in retrograde endosome-Golgi trafficking, VPS35 may also be involved in regulation of exosomal secretion [62]. In support of previous findings [48], [63], we also report the presence of APP, C-terminal fragments and BACE1 in secreted exosomes. Our multiplex assay system offers a novel, highly sensitive tool to study the dynamics of exosomal secretion of APP and BACE1. In general, parallel assessment of multiple pathways may provide new insight into complex subcellular trafficking itineraries of APP. For example, sphingolipid metabolism modulates trafficking and processing of APP and BACE1 on multiple levels. Using the multiplex APP PCA, the strong effects of nSMase inhibition by GW-4869 on both shedding and exosomal secretion of APP were revealed, suggesting the involvement of ceramide in both processes.

There are two obvious caveats with the multiplex APP assay. First, analysis of sAPP is not specific to the sAPP-β form but detects both the sAPP-α and sAPP-β forms. Using a mutation near the α-cleavage site to block α-secretase cleavage is an attractive strategy but this would require near-full inhibition, which cannot be achieved with the F615P mutation [40], [41]. Secondly, the assay requires overexpression of APP and BACE1. Although this may alter the natural trafficking and processing machinery or the cellular localization of the reporters to some degree, it should be noted that most cell lines and transgenic animal models used in AD research similarly rely on overexpression of APP. Currently, the assay relies on transient transfection but for more extensive screening studies better control of the overexpression level of the reporter proteins would require generation of a stable reporter cell line.

Sporadic forms of neurodegenerative diseases are complex and their etiologies cannot be narrowed down to a small number of effector genes. Particularly, in the case of late-onset Alzheimer's disease (LOAD), which comprises more than 95% of the AD patient population, the functional connections of the risk genes to the known pathophysiological pathways of the disease remains poorly understood. In the post-GWAS-era, a major bottleneck is functional characterization of novel disease-associated genes. Cell-based assays that facilitate rapid screening of functional connections of novel disease-associated genes to known pathophysiological pathways of the disease could significantly accelerate this process. Current methods used to study APP-related aspects of AD pathogenesis mostly rely on analyzing protein complexes in cell or tissue extracts. Live-cell methods are more suitable for studying the dynamics of cellular trafficking of disease-associated proteins and their protein interactions, and may reveal new subtle nuances in cellular regulation of proteins of interest, while providing high-throughput abilities. Our multiplex live-cell assay provides a sensitive and dynamic reporter system that responds predictably to stimuli known to modulate amyloidogenic processing of APP in cells. This offers a novel and rapid way of addressing mechanistic aspects of e.g. altered amyloidogenic processing of APP in cells. Therefore, our novel assay platform offers significant advantages for various screening approaches, from drug discovery to functional characterization of late-onset AD risk genes.
